# Vitamin D-Mediated Immunoregulation in Degenerative Diseases: Insights into Cardiovascular, Neurodegenerative and Musculoskeletal Disorders

**DOI:** 10.3390/nu18040629

**Published:** 2026-02-14

**Authors:** Ga Young Lee, Chan Yoon Park, Sung Nim Han

**Affiliations:** 1 Department of Food and Nutrition, College of Human Ecology, Seoul National University, Seoul 08826, Republic of Korea; lgykiki90@snu.ac.kr; 2 Department of Food & Nutrition, College of Life Care Science Technology, The University of Suwon, Hwaseong-Si 18323, Republic of Korea; 3 Research Institute of Human Ecology, Seoul National University, Seoul 08826, Republic of Korea

**Keywords:** vitamin D, immunomodulation, neurodegeneration, osteoporosis, sarcopenia, cardiovascular diseases

## Abstract

Degenerative diseases are characterized by the gradual loss of cellular integrity, tissue function, and regenerative capacity. Cardiovascular diseases, neurodegenerative disorders, and musculoskeletal deterioration are considered major categories of degenerative diseases, and vitamin D deficiency has been linked with an increased risk of these conditions. Vitamin D has the potential to modulate neurogenerative process by influencing the progression of neuronal survival, neurogenesis, and synaptic plasticity through both genomic and non-genomic mechanisms mediated by vitamin D receptors, which are widely distributed across brain regions and cell types. Additionally, vitamin D regulates brain immunometabolism by modulating microglial and astrocytic inflammatory responses and oxidative stress. Vitamin D has long been recognized as essential for bone health. Beyond its classical role, vitamin D contributes to the maintenance of bone–muscle homeostasis, enhances mitochondrial biogenesis and ATP production while reducing oxidative stress, and facilitates bidirectional bone–muscle crosstalk through myokines and osteokines to coordinate bone remodeling and muscle regeneration. However, despite these mechanistic insights, the beneficial effects of vitamin D on these diseases—such as reduced risk or mitigation of progression—remains inconclusive. This review explores the relationships between vitamin D and cardiovascular, neurodegenerative, and musculoskeletal diseases, with a focus on the underlying immunological and metabolic mechanisms of actions.

## 1. Introduction

Degenerative diseases comprise a broad group of chronic, progressive disorders characterized by the gradual loss of cellular integrity, tissue function, and regenerative capacity. These conditions, including cardiovascular diseases, neurodegenerative disorders, and musculoskeletal deterioration, are major contributors to morbidity, disability, and mortality in aging populations worldwide. Although these diseases affect distinct organ systems, they share common mechanistic pathways driven by chronic low-grade inflammation, immune dysregulation, oxidative stress, and mitochondrial dysfunction. Understanding the molecular factors that modulate these processes is crucial for developing effective preventive and therapeutic strategies.

Vitamin D exerts its biological effects through a complex metabolic cascade involving metabolites, 25-hydroxyvitamin D (25(OH)D) and its active form, 1,25-dihydroxyvitamin D (1,25(OH)_2_D). These actions are mediated primarily through the vitamin D receptor (VDR), which is expressed across many different cells of cardiovascular system, brain, skeletal muscle, and bone. This widespread VDR distribution underlies the pleiotropic actions of vitamin D, extending beyond its classical endocrine role in calcium and phosphate homeostasis to metabolic effects, including the regulation of oxidative stress and mitochondrial function, as well as immune and inflammatory regulation and tissue remodeling [1,2].

At the cellular level, vitamin D regulates target cell function through genomic and non-genomic mechanisms. Genomic actions are mediated by the binding of 1,25(OH)_2_D to VDR, which leads to transcriptional regulation of target genes. In contrast, rapid nongenomic signaling is mediated by membrane-associated VDR or by protein disulfide isomerases A3 (PDIA3), also known as membrane-associated, rapid response steroid-binding (MARRS). In addition, the presence of vitamin D-metabolizing enzymes, including *Cyp27a1* (25-hydroxylase), *Cyp27b1* (1-hydroxylase), and *Cyp24a1*(24-hydroxylase), in extra-renal tissues suggests the possibility of local bioactivation of vitamin D at the tissue level [3,4,5].

Furthermore, vitamin D contributes to the maintenance of immune homeostasis in part through the modulation of mitochondrial function and oxidative stress by improving mitochondrial dynamics while decreasing generation of reactive oxygen species (ROS) [6]. These mechanisms collectively enhance the intracellular environment of muscle cells and mitigate tissue damage [6]. Although the precise molecular mechanisms have not yet been fully elucidated, vitamin D has been shown to regulate key pathways involved in mitochondrial biogenesis and redox homeostasis, including peroxisome proliferator-activated receptor gamma coactivator-1α (PGC-1α), forkhead box O (FOXO) transcription factors, and sirtuin-1 (SIRT-1) [7,8]. These pathways are closely associated with cellular metabolism, inflammatory regulation, and aging-related cellular dysfunction [9,10,11].

Vitamin D insufficiency and deficiency are highly prevalent in ageing populations due to reduced cutaneous synthesis, inadequate dietary intake, and age-related changes in vitamin D metabolism [12]. However, the definition of vitamin D deficiency varies across the scientific literature. The Institute of Medicine (IOM) defines serum 25(OH)D levels < 12 ng/mL as indicating a clear risk to bone health, whereas levels of 12–20 ng/mL are considered potentially inadequate for some individuals, based on conservative criteria that emphasize causal relationships with skeletal outcomes [13]. In contrast, the 2011 Endocrine Society clinical practice guideline classifies serum 25(OH)D levels < 20 ng/mL as deficient and levels < 30 ng/mL as insufficient [14]. More recently, updated recommendations published in 2024 highlighted insufficient evidence to support universal vitamin D supplementation in the general population and instead recommended a targeted approach for those with clear indications, such as older adults and those with obesity or chronic diseases [15].

Studies have shown that age-related declines in vitamin D status are associated with an increased risk of chronic diseases, including cardiovascular disorders, neurodegenerative diseases, osteoporosis, sarcopenia [16,17]. Although cardiovascular, neurodegenerative, and musculoskeletal disorders represent biologically distinct conditions, they share several common pathological features, including chronic low-grade inflammation, immune dysregulation, oxidative stress, and age-associated functional decline. Accordingly, these disease categories are discussed together in the present review based on their shared features, while disease-specific processes are addressed in the respective sections.

Vitamin D is known to interact with other fat-soluble vitamins, particularly vitamins A and E, through competitive interactions that may modify its biological effects [18]. Although such interactions may contribute to variability in vitamin D-related outcomes, accumulating evidence suggests that immunomodulatory and inflammatory pathways are key mechanisms through which vitamin D influences the process of aging-related diseases [19,20]. Taken together, this review focuses on the immune and inflammatory pathways related to vitamin D and discusses its potential roles in the prevention and progression of cardiovascular, neurodegenerative, and musculoskeletal diseases.

## 2. Cardiovascular Diseases

Vitamin D influences cardiovascular physiology and pathology through several different mechanisms affecting various cell types. Cells of the cardiovascular systems, such as endothelial cells, vascular smooth muscle cells (VSMCs), and cardiac cells, have been reported to be affected by vitamin D. Because these cells are essential for maintaining vascular homeostasis, regulating vascular tone, angiogenesis, and blood pressure, vitamin D can modulate cardiovascular function and progression of cardiovascular diseases (CVD) [21].

### 2.1. Vitamin D and Endothelial Cells

Dysfunction of vascular endothelium is linked to the pathogenesis of CVD and has been observed with vitamin D deficiency. Serum levels of endothelial-specific molecule-1 (endocan), which has been associated with endothelial dysfunction and acute myocardial infarction, were significantly higher in adults aged 18–45 years with low vitamin D levels (<10 ng/mL). An inverse linear relationship was observed between serum endocan concentrations and vitamin D levels [22]. Additionally, endothelial-specific VDR-knock out mice showed impaired acetylcholine-induced aortic relaxation, reduced nitric oxide (NO) synthase expression, and increased sensitivity to the hypertensive effects of angiotensin II, highlighting the importance of vitamin D in endothelial cell function [23]. Several studies have reported improved endothelial function with vitamin D. In vitro treatment of endothelial cells with 25(OH)D or 1,25(OH)_2_D showed decreased expression of adhesion molecules such as intercellular adhesion molecule (ICAM) and vascular cell adhesion molecule (VCAM), interleukin (IL)-6 expression and secretion, tissue factor expression, oxidative and endoplasmic reticulum stress, and increased nitric oxide (NO) production [21,24]. In a meta-analysis study [25], vitamin D supplementation was reported to improve flow-mediated dilation, a marker of arterial dilation in response to increased blood flow, and pulse wave velocity, a measure of arterial stiffness; however, did not show significant effect on the augmentation index, a measure of arterial wave reflection. The beneficial effects of vitamin D were mostly observed in individuals with vitamin D deficiency, such as patients with chronic kidney diseases (CKD) who exhibit decreased vitamin D activation.

### 2.2. Vitamin D and Vascular Smooth Muscle Cells

Vitamin D modulates pro-inflammatory signaling in vascular tissues, including toll-like receptor (TLR) 4-mediated pathways, IL-6 production, and monocyte chemoattractant protein (MCP)-1 expression, which affect VSMC function in atherosclerosis-related processes. VSMCs, which comprise the medial layer of arteries, play an important role in regulating vascular function [26]. Phenotypic changes in VSMCs, such as enhanced rate of proliferation and production of extracellular matrix components, can contribute to CVD. Additionally, the formation of VSMC-derived foam cells and the migration of VSMCs into the intima are important drivers of atherosclerosis [21,27]. Vitamin D treatment has been shown to suppress oxidized (ox)-LDL-mediated VSMC foam cell formation, which appears to be linked to inhibition of TLR4 signaling; TLR4 signaling is a key regulator of VSMC-derived foam cell formation and vitamin D inhibits the upregulation of TLR4 in response to ox-LDL, as well as MyD88, IL-6, and MCP-1 [27]. However, excessive vitamin D has been linked to an increased risk of vascular calcification, thereby augmenting susceptibility to cardiovascular events [28]. Chronic kidney disease (CKD) is also associated with vascular calcification, which has been described in some studies as vascular ossification. This term reflects an active, cell-mediated process resembling osteogenic differentiation rather than passive mineral deposition [29,30]. Aortic smooth muscle cells treated with 1,25(OH)_2_D showed phenotypic and growth pattern changes associated with atherosclerosis [31]. Furthermore, high-dose vitamin D administration (7.5 mg/kg) in rats induced arterial calcium and phosphorus accumulation and matrix increased metalloproteinase (MMP)-9 expression [32]. In contrast, a community-based cohort study found no association between levels of vitamin D metabolites and coronary artery calcification (CAC) among 5945 participants, with 50% of participants exhibiting prevalent CAC [33].

### 2.3. Vitamin D and the Incidence of Cardiovascular Disease

Several observational studies have reported that low vitamin D levels are associated with a higher incidence of CVD [34]. However, interventional studies have not shown conclusive beneficial effects of vitamin D supplementation on the incidence of CVD. Vitamin D levels have been reported to be associated with all-cause mortality in patients with a history of myocardial infarction in a U-shaped manner [35]. Both low and high plasma 25(OH)D concentrations were associated with higher mortality. Patients with 25(OH)D levels lower than 10 ng/mL and above 30 ng/mL showed cumulative survival rates of 66% and 79%, respectively, after 5 years, compared with survival rates of 88% and 90% in those with concentrations of 10–20 ng/mL and 20–30 ng/mL, respectively. While vitamin D deficiency is associated with increased risks of cardiovascular and other diseases, excessively high levels may be harmful due to hypercalcemia, vascular calcification, and alterations in immune function, providing a plausible biological basis for a U-shaped relationship. Clinically, this suggests that supplementation should aim to correct deficiency rather than achieve very high serum concentrations, with careful monitoring to prevent potential adverse effects.

## 3. Neurodegeneration

### 3.1. Neuroprotective and Immunometabolic Roles of Vitamin D in the Brain

Accumulating evidence suggests an association between vitamin D status and neurodegenerative diseases. Low serum 25(OH)D levels, indicative of vitamin D deficiency, have been recognized as a potential risk factor for Alzheimer’s disease (AD), Parkinson’s disease (PD), and other neurodegenerative diseases. Several animal studies, including dietary vitamin D deficiency and VDR knockdown models, have demonstrated that vitamin D deficiency can exacerbate the progression of neurodegenerative disorders. Furthermore, vitamin D promotes neuronal function and neuroprotection via neurotrophic factors and by regulating brain immune cells through immunometabolic pathways, through both genomic and nongenomic mechanisms. These effects involve disease-specific immune pathways, including microglial activation and astrocyte-mediated inflammatory responses, which are critical in the pathogenesis of AD, PD, and other neurodegenerative diseases.

(1) Genomic and non-genomic actions of vitamin D and VDR distribution in the brain

Accumulating evidence has demonstrated that VDR is expressed across multiple brain regions, including the hippocampus, prefrontal cortex, substantia nigra, thalamus, and cerebellum, as well as in various cell types such as neurons, astrocytes, endothelial cells, and microglia, with particularly high expression in astrocytes [36,37,38,39,40,41]. These findings suggest that astrocytes may be potential targets of the genomic actions of calcitriol [42].

VDR expression has been detected during gestation and early development, suggesting a potential role for embryonic VDR in regulating neuronal differentiation, proliferation, and programmed cell death [43]. In animal models, maternal vitamin D deficiency has been associated with reduced levels of neurotrophic factors, including nerve growth factor (NGF) and glial cell line-derived neurotrophic factor (GDNF), in the brains of offspring, indicating the importance of adequate maternal vitamin D for normal neurodevelopment [44].

Growing evidence indicates that vitamin D metabolites can cross the blood–brain barrier (BBB) and directly interact with the VDR in the brain, either as 25(OH)D or 1,25(OH)_2_D. Through these interactions, vitamin D modulates a range of central nervous system (CNS) functions, including neuroinflammatory responses, oxidative stress regulation, and neurotrophic factor expression [45]. It has been suggested that the availability of 1,25(OH)_2_D in the brain depends on circulating plasma concentration [46,47]. However, its precursor form, cholecalciferol (vitamin D3), can also cross the BBB and may exert region-specific effects through local bioactivation, as the enzymes required for its conversion are expressed not only in the liver and kidney but also in pericytes, glial cells, and neurons.

(2) Neuroprotective effect of vitamin D on neuron

Vitamin D contributes to neuroprotection by regulating neuronal and microglial differentiation and proliferation, dopamine signaling, and neurotrophic factor production, thereby enhancing neuronal survival and neuroplasticity as demonstrated in in vitro, ex vivo, and animal studies [48,49]. Vitamin D promotes NGF synthesis, neurite outgrowth, and cytoskeletal stabilization while reducing excessive cell proliferation [50,51]. 1,25(OH)_2_D treatment with cultured embryonic hippocampal cells and explants have been shown to significantly reduce the proportion of cells undergoing mitosis, while increasing neurite outgrowth and NGF production [50]. Treatment of neural stem cells with 1,25(OH)_2_D enhances cell survival by upregulating neurotrophic factors, including neurotrophin-3 (NT-3), brain-derived neurotrophic factor (BDNF), GDNF, and ciliary neurotrophic factor (CNTF), and promotes differentiation into neurons and oligodendrocytes, with no effect on astrocytes [52]. Although 1,25(OH)_2_D does not appear to directly promote astrocytic differentiation, vitamin D helps modulate glial function and neuron–glia interactions, thereby indirectly supporting neuronal survival, synaptic stability, and resistance to neurodegenerative stress [53]. 1,25(OH)_2_D upregulates NGF, NT-3, and GDNF, while down-regulating neutrophin-4 (NT-4) in glioma cell lines, and promotes neurite outgrowth in cultured hippocampal cells [5,54]. In cultured rat cortical neurons, 1,25(OH)_2_D has been reported to protect against excitotoxicity by upregulating proteins essential for axonal growth and synaptic function, thereby enhancing neuronal plasticity through cytoskeletal stabilization [51].

Conversely, vitamin D deficiency has been reported to impair neurogenesis and brain development. Rats exposed to vitamin D deficiency until birth or weaning showed enlarged lateral ventricles, lower NGF levels, and reduced expression of genes related to neuronal structure (e.g., neurofilament and MAP2) and neurotransmission (GABA-A α4) [55]. Maternal vitamin D supplementation resulted in an increase in the number of neurospheres derived from the neonatal subventricular zone, suggesting enhanced proliferative activity in the developing brain [56]. However, supplementation with exogenous vitamin D normalized proliferation in control cultures but did not completely reverse the effects observed under vitamin D-deficient conditions [56]. Similarly, vitamin D deficiency during gestation has been reported to promote cell proliferation and reduce apoptosis across multiple brain regions, resulting in abnormal central nervous system development [57].

Vitamin D has also been implicated in the regulation of dopaminergic neurogenesis and differentiation, with developmental vitamin D deficiency being linked to neurodevelopmental disorders characterized by abnormal dopamine signaling, such as schizophrenia [58]. Overexpression of VDR in both undifferentiated and differentiated SH-SY5Y cells enhances dopamine production and upregulates the expression of tyrosine hydroxylase and catechol-O-methyltransferase (COMT), key enzymes involved in dopamine synthesis and metabolism [59,60]. Moreover, vitamin D confers neuroprotective effects by inducing Ca^2+^-binding proteins such as parvalbumin [61], and by inhibiting inducible NO synthase, which is upregulated in neurodegenerative conditions including AD and PD [62].

(3) Immunomodulatory and anti-inflammatory effects of vitamin D on immune cells in the brain

Vitamin D modulates immune and inflammatory response. In monocytes and macrophages, 1,25(OH)_2_D consistently reduces the expression and production of pro-inflammatory cytokines [63,64,65,66]. One proposed mechanism underlying these effects involves modulation of Toll-like receptors (TLRs). Vitamin D has been reported to reduce protein and mRNA expression levels of TLR2 and TLR4 in human monocytes in a time- and dose-dependent manner, followed by decreased production of pro-inflammatory cytokines such as IL-6 and tumor necrosis factor (TNF)-α [63,64,65]. Vitamin D also suppresses inflammatory responses by targeting the nuclear factor κB (NF-κB) and mitogen-activated protein kinase (MAPK) signaling pathways [67]. Specifically, 1,25(OH)_2_D inhibits NF-κB activation by stabilizing IκBα, thereby preventing nuclear translocation of the p65/p50 complex. This effect may be mediated, at least in part, by enhanced interaction between the VDR and IκB kinase (IKK), thereby limiting NF-κB signaling [68].

In the brains of aged rodents, levels of IL-1β and IL-6 are increased, whereas IL-10 and IL-4, which are anti-inflammatory cytokines, are reduced [69]. Microglia, which constitute 16% of cells in the CNS, are the resident macrophages of the brain and play essential roles in development, homeostasis, and neuroinflammation [69]. Aging is associated with a shift in microglial phenotype toward a pro-inflammatory state, accompanied by reduced phagocytic capacity, increased reactive oxygen species (ROS) production, enhanced secretion of pro-inflammatory cytokines, and upregulated expression of inflammatory receptors [70]. These age-related alterations in microglial function have been increasingly linked to neurological dysfunction.

Vitamin D_3_ modulates immune responses, particularly within the brain, by acting on microglia and astrocytes. Interferon (IFN)-γ- stimulated microglia shows increased expression of VDR and *Cyp27b1*, indicating that inflammatory signals enhance microglial responsiveness to vitamin D_3_ [71]. In the presence of 1,25(OH)_2_D, IFN-γ- or LPS-stimulated microglia exhibit reduced expression of pro-inflammatory cytokines and increased expression of the anti-inflammatory cytokine IL-10 [71]. Furthermore, 1,25(OH)_2_D dose-dependently inhibits Staphylococcal enterotoxin B-induced TNF production in microglial cells [72].

In an in vivo mouse model of PD, vitamin D administration attenuated the expression of inducible nitric oxide synthase (iNOS) and TLR4, while increasing expression levels of IL-10, IL-4, and TGF-β and promoting an M2 microglial phenotype (CD163, CD206, and CD204) [73]. Consistent with these findings, vitamin D_3_ suppresses the expression of TNF-α, IL-1β, vascular endothelial growth factor (VEGF), and TLR4 in LPS-stimulated astrocytes [74]. Moreover, vitamin D_3_ treatment protects astrocytes against rotenone-induced cellular damage by reducing oxidative stress and downregulating NF-kB and NRF2 signaling pathways [75].

### 3.2. Neurodegenerative Diseases: Biological Mechanisms and Clinical Evidence Related to Vitamin D

#### 3.2.1. Alzheimer’s Disease

AD is the most common form of dementia and is characterized by progressive cognitive decline. It is estimated that over 55 million people worldwide live with dementia, including AD, and this number is projected to increase to approximately 139 million by 2050s [76]. The World Health Organization has predicted that neurodegenerative diseases, including AD and PD, will become one of the leading causes of death globally by 2040, surpassing cancer-related mortality [77,78]. The pathophysiology of AD is characterized by the accumulation of amyloid-beta(Aβ) peptides and the formation of amyloid plaques, which precede tau pathology, neuronal loss, and the onset of clinical symptoms. Aβ is generated through the sequential cleavage of amyloid precursor protein (APP) by β-secretase (BACE1) and γ-secretase (presenilin-1, PS1) [79]. The aggregation and accumulation of Aβ around neurons induces neurotoxicity, triggers inflammatory responses, and ultimately leads to neuronal death [80]. Although Aβ deposition is a hallmark feature of AD, early pathological changes, such as neuroinflammation and atrophy of the hippocampus and specific brain regions, have also been reported [81,82].

*(1) Mechanisms and* *in vitro and in vivo studies*

Numerous in vitro studies using neuronal and glial cells, as well as in vivo studies employing AD models, have investigated whether vitamin D influences AD pathogenesis. These studies indicate that vitamin D exerts beneficial effects by reducing Aβ accumulation and inflammatory responses, enhancing antioxidant defenses, and increasing the production of neurotrophic factors.

Regarding Aβ pathology, vitamin D and its analogues have been shown to reduce Aβ burden by both suppressing Aβ production and enhancing its clearance. These effects have been attributed to modulation of the expression and activity of BACE1 and PS1 in neuroblastoma cells and in the brains of vitamin D-deficient mice [83]. Additionally, short- and long-term treatment with 1,25(OH)_2_D_3_ in AD model mice increased brain P-glycoprotein (P-gp) levels, reduced soluble Aβ levels, and improved conditioned fear memory. In mice fed a vitamin D-deficient diet, cerebral P-gp expression was reduced, but was restored upon supplementation [84]. In transgenic AD-like mice (5XFAD model), high-dose vitamin D supplementation improved working memory and neurogenesis, while early vitamin D deficiency exacerbated amyloid plaque deposition [85]. Moreover, VDR activation attenuated tau phosphorylation, potentially through inhibition of glycogen synthase kinase 3β (GSK3β) activity in APP/PS1 mice [86].

Moreover, the neuroprotective and anti-inflammatory effects of vitamin D in neurons, astrocytes, and microglia, as described in “3.1. Neuroprotective and Immunometabolic Roles of Vitamin D in the Brain”, may further mitigate AD-related neuroinflammation, thereby contributing to the overall neuroprotective impact of vitamin D in AD pathogenesis

*(2) Clinical studies*

Growing evidence indicates that vitamin D status is associated with the risk of cognitive decline and dementia (Table 1). Observational studies have reported associations between vitamin D deficiency and an increased risk of dementia, particularly AD [87,88]. Meta-analyses of prospective cohort studies further support this association, showing that individuals with low vitamin D levels have 1.3–1.8 folds higher risk of all-cause dementia and AD compared with those with sufficient levels [89,90,91]. In a meta-analysis of 23 prospective cohorts, vitamin D deficiency was associated with a 42% increased risk of dementia (95% CI: 1.21–1.65) and a 57% higher risk of AD (95% CI: 1.15–2.14) [90]. Recent observational and Mendelian randomization (MR) analyses have revealed nonlinear associations between serum 25(OH)D levels and total brain, gray matter, white matter, and hippocampal volumes [92]. Notably, serum 25(OH)D levels below 25 nmol/L were associated with the highest dementia risk, and MR analyses suggested a potential causal contribution of vitamin D deficiency to dementia, but not in stroke. Collectively, these findings support an association between vitamin D deficiency and AD-related outcomes, while a direct casual role in AD pathogenesis remains uncertain.

Furthermore, several clinical studies have explored the effect of higher dietary vitamin D intake on AD risk. A 7-year longitudinal study reported that individuals in the highest quintile of vitamin D intake had a significantly lower risk of developing AD compared with those in the lower four quintiles [100]. However, findings from intervention studies remain inconsistent [101]. While some trials suggest that vitamin D supplementation may attenuate cognitive decline and normalize Aβ levels [102], others have reported no significant cognitive benefits [103]. Notably, several studies have even raised concerns that vitamin D supplementation may exacerbate AD progression under certain conditions [104,105]. For example, a population-based longitudinal study found that dementia-free older adults (*n* = 14,648) who consumed vitamin D_3_ supplements for more than 146 days per year were 1.8 times more likely to develop dementia compared with non-supplement users [104]. While, a 6-month supplementation trial in patients with mild cognitive impairment (MCI) and very early AD (VEAD) showed improvements in oxidative stress resistance, plasma Aβ1–40 levels, and cognitive performance in MCI patients, but not in those with VEAD, possibly reflecting differences in disease stage [102]. Consistent with these mixed findings, a meta-analysis of nine RCTs involving 2345 participants found no significant differences between vitamin D and control groups across multiple AD-related cognitive outcomes, such as Mini-Mental State Examination scores, verbal fluency, verbal memory, visuospatial ability, and attention [99]. Collectively, although low vitamin D status is associated with an increased risk of cognitive decline and dementia, the efficacy of vitamin D supplementation as a preventive or therapeutic strategy for AD remains inconclusive.

#### 3.2.2. Parkinson’s Disease

PD is a progressive chronic neurodegenerative disorder characterized by both motor symptoms—including rigidity, tremor, and gait disturbances -and non-motor symptoms, such as constipation, neuropathy, pain, sleep disorders, and cognitive decline. The global prevalence of PD is projected to reach 25.2 million individuals by 2050, representing an estimated 112% increase compared with 2021 levels [78]. A defining pathological feature of PD is the progressive degeneration of dopaminergic neurons in the substantia nigra pars compacta (SNpc), which is closely linked to the emergence of motor symptoms. Another hallmark of PD is Lewy pathology, characterized by the accumulation of misfolded α-synuclein in the form of Lewy bodies and Lewy neurites. The aggregation of aberrantly folded proteins is increasingly recognized as a shared pathological mechanism across neurodegenerative disorders, including PD [106]. In addition to these hallmark features, PD-related neurodegeneration has been linked to multiple interconnected processes, such as excitotoxicity, apoptosis, oxidative stress, mitochondrial dysfunction, and neuroinflammation [107].

*(1) Mechanisms and* *in vitro and in vivo studies*

VDRs are widely distributed throughout the brain and are particularly enriched in the nigrostriatal tract and motor cortex, suggesting a potential involvement of vitamin D signaling in PD pathophysiology [108,109]. Given that the nigrostriatal tract is the primary site of neurodegeneration in PD, the high density of VDRs in this region further supports a functional role for vitamin D in maintaining dopaminergic neuron integrity.

Experimental studies suggest that vitamin D may promote the recovery of dopaminergic function in injured nigrostriatal neurons, partly through the modulation of autophagy, while also providing protection against neuroinflammation and oxidative stress in hemiparkinsonian rat models [110,111]. The vitamin D analog calcipotriol has been shown to inhibit α-synuclein aggregation and attenuate PD-related pathology by increasing intraneuronal Ca^2+^ buffering capacity via transcriptional upregulation of calbindin-D28k in SH-SY5Y neuroblastoma cells [112,113].

Consistent with these findings, treatment with 1,25(OH)_2_D has been reported to increase GDNF expression and mitigate the loss of tyrosine hydroxylase immunoreactivity in the substantia nigra of PD model rats, indicating its potential to protect dopaminergic neurons [114]. Moreover, vitamin D supplementation in PD-induced mice has been shown to reverse 6-hydroxydopamine (6-OHDA)-induced alterations in dopamine-regulating proteins, behavioral impairments, and oxidative stress markers. These findings suggest that vitamin D_3_ may help lower the required dose of levodopa (L-DOPA), potentially mitigating its adverse effects [110]. In Klotho-deficient mice, aberrant activation of vitamin D signaling was associated with age-related declines in striatal dopamine levels and reductions in TH-positive neurons in the substantia nigra and ventral tegmental area. Notably, these dopaminergic deficits were reversed by dietary vitamin D restriction [115]. These findings suggest that dysregulated vitamin D activation resulting from Klotho insufficiency may contribute to dopaminergic neuronal loss. In addition to its direct effects on dopaminergic neurons, vitamin D exerts anti-inflammatory and antioxidant actions through VDR-mediated signaling, supporting its potential role in the prevention and adjunctive treatment of PD [116].

*(2) Clinical studies*

A high prevalence of vitamin D deficiency has been reported among patients with PD (Table 2). Multiple studies indicate that vitamin D deficiency or insufficiency is common in individuals with PD [117]. Moreover, serum 25(OH)D concentrations are significantly lower in patients with PD than in those with AD or healthy controls, suggesting a disease-specific association [118,119].

Clinically, lower serum vitamin D levels have been associated with greater motor symptom severity in PD [127]. Several interventional studies suggest that high-dose vitamin D supplementation may improve balance in younger patients with PD and modestly enhance functional outcomes, such as performance on the 6 min walking test and certain motor symptoms, indicating a potential modulatory role of vitamin D in PD progression [113]. Furthermore, a meta-analysis of eight clinical studies revealed that vitamin D insufficiency or deficiency, as well as reduced sunlight exposure, was significantly associated with an increased risk of PD; however, vitamin D supplementation did not lead to significant improvements in motor function in patients with PD [117]. However, a prospective cohort study reported no significant difference in serum vitamin D levels between individuals at high and low risk of PD [128]. These findings suggest that the association between vitamin D status and PD susceptibility may vary according to disease stage, population characteristics, or the influence of confounding factors.

#### 3.2.3. Other Neurodegenerative Diseases

*(1) Multiple sclerosis*

Multiple sclerosis (MS) is an auto-immune and neurodegenerative disease characterized by demyelination and inflammation of the CNS, which disrupts normal signal transmission between the brain and peripheral tissues. It commonly presents with an acute CNS inflammatory event—such as optic neuritis, transverse myelitis, or brainstem syndromes [129]. Although MS is a complex disease with diverse clinical and pathological phenotypes, autoimmune T-cell-mediated inflammation represents a central mechanism that initiates axonal and myelin injury and may subsequently contribute to immune-independent neurodegeneration [130]. Several factors, including female sex, smoking, viral infections, hormonal imbalance, and vitamin D deficiency, have been associated with an increased risk of MS [129,131,132].

Vitamin D has emerged as a biologically plausible factor influencing MS risk and disease activity, supported by its immunoregulatory properties. In experimental autoimmune encephalomyelitis (EAE), a widely used animal model of MS, vitamin D supplementation and administration of the active metabolite 1,25(OH)_2_D have been shown to delay disease onset and attenuate disease progression. These effects are mediated by reductions in infiltrating CD11b^+^ and CD4^+^ immune cells, decreased production of pro-inflammatory cytokines, and a shift toward an anti-inflammatory profile with increased IL-10 and IL-4 secretion [133,134]. Mechanistically, 1,25(OH)_2_D promotes regulatory T-cell differentiation and helps maintain the balance between Th1/Th2 lymphocyte subsets, thereby contributing to disease amelioration [135]. Consistent with these findings, vitamin D supplementation in patients with MS for 6 months has been reported to increase transforming growth factor (TGF)-β1 production [136], supporting its therapeutic relevance in MS. 

Historically, epidemiologic studies have consistently shown that the prevalence of MS is higher at greater latitudes with lower ultraviolet (UV) exposure, whereas diets rich in vitamin D (e.g., oily fish) may partially mitigate this increased risk [137,138,139,140]. Observational studies have also consistently reported an inverse association between circulating vitamin D levels and MS risk. A longitudinal study of U.S. military personnel showed that individuals with serum 25(OH)D levels ≥ 100 nmol/L had a substantially lower risk of developing MS [131]. A recent systematic review similarly reported a reduced risk of MS among participants using vitamin D supplements [141]. Mendelian randomization analyses further support a causal relationship between genetically determined low 25(OH)D levels and increased MS susceptibility, thereby reducing concerns regarding confounding and reverse causation inherent to observational studies [142,143]. More recently, the randomized, double-blind, placebo-controlled D-Lay MS trial demonstrated that high-dose cholecalciferol (100,000 IU every 2 weeks) significantly reduced overall disease activity in patients with clinically isolated syndrome and early relapsing-remitting MS [129]. Although some studies showed no significant effects, the overall body of evidence supports vitamin D deficiency as a modifiable risk factor for MS and suggests potential benefits of vitamin D supplementation in early disease stages [144].

*(2) Amyotrophic lateral sclerosis*

Amyotrophic lateral sclerosis (ALS), also known as Lou Gehrig’s disease, is a fatal progressive neurodegenerative disease of unknown etiology that selectively affects upper and lower motor neurons, resulting in muscle weakness and paralysis. Accumulating evidence indicates ALS pathogenesis involves multiple interrelated mechanisms, including oxidative stress, neuroinflammation, mitochondrial dysfunction, impaired axonal transport, and genetic factors [145]. Vitamin D has been shown to reduce oxidative stress and inflammatory mediators (TNFα, IL-1β, and NOS), while increasing the expression of neuroprotective factors, which may indicate a potential neuroprotective role in ALS, as suggested in other neurodegenerative diseases [145]. Furthermore, vitamin D may be involved in modulating processes associated with the pathogenesis and progression of ALS through both genomic and non-genomic mechanisms. Genomically, it suppresses the expression of MHC class II antigens and TLRs (TLR2, TLR4, and TLR9), whereas non-genomic actions include reductions in glutamate-induced neurotoxicity and matrix metalloproteinase activity [146].

## 4. Musculoskeletal Disorders

### 4.1. Vitamin D-Mediated Immune Regulation in Bone and Muscle

(1) Modulation of immune cell function and cytokine profiles by vitamin D in bone and muscle

Binding of 1,25(OH)_2_D on VDR expressed on monocytes, macrophages, and lymphocytes activates monocytes and macrophages to enhance host defense mechanisms, while inducing immunosuppressive responses in lymphocytes, characterized by reduced T and B cell activity [147]. Vitamin D has been shown to suppress dendritic cell maturation and differentiation, sustaining an immature phenotype with reduced IL-12 and MHC class II expressions. This mechanism is essential for preventing excessive immune activation and inflammatory responses, thereby maintaining immune homeostasis [148]. Furthermore, vitamin D modulates T cell differentiation by enhancing Treg formation and suppressing Th17 cell activity, resulting in a shift toward an anti-inflammatory cytokine profile that supports bone and muscle homeostasis [149]. In skeletal muscle tissue, vitamin D regulates immune and inflammatory responses by attenuating excessive inflammation and facilitating immune surveillance processes essential for muscle regeneration [150]. These findings indicate that, in bone and muscle, vitamin D modulates macrophage-driven bone resorption and enhances regulatory T cell–mediated anti-inflammatory responses, both of which are critical for maintaining musculoskeletal homeostasis.

Vitamin D suppresses monocyte-derived pro-inflammatory cytokines, including TNF-α, IL-1β, and IL-6 as shown in both cell-based and animal studies, which is linked to the amelioration of inflammatory conditions and bone loss [149]. Consistent with these findings, calcitriol supplementation in postmenopausal women with osteoporosis has been reported to reduce IL-1 and TNF-α levels while increasing bone mineral density (BMD) [147].

(2) Regulation of intracellular signaling within bone and muscle cells by vitamin D

Vitamin D regulates important cellular processes such as inflammatory responses, differentiation, proliferation, and apoptosis across osteoblasts, osteoclasts, and muscle cells through VDR-mediated genomic and non-genomic actions. It has been reported that 1,25(OH)_2_D promotes osteoclastogenesis by upregulating receptor activator of nuclear factor-κB ligand (RANKL) expression in osteoblasts in vitro [151]. However, in vivo, vitamin D enhances intestinal calcium absorption, accompanied by improved systemic calcium homeostasis [152]. This leads to suppression of parathyroid hormone (PTH) secretion, which in turn attenuates RANKL-mediated bone resorption signaling in osteoblasts and osteocytes, ultimately resulting in an inhibitory effect on bone resorption [152]. These alterations in calcium homeostasis and PTH-RANKL-mediated signaling contribute to the maintenance of bone remodeling balance [147].

Vitamin D is also known to promote protein kinase C (PKC) activation and promote cytosolic calcium release [153]. These effects mediate active calcium sequestration into the sarcoplasmic reticulum via calcium ATPase (Ca-ATPase), which is essential for muscle contraction. Additionally, PKC activation has been shown to regulate protein synthesis in muscle cells [154], highlighting its critical role in maintaining muscle cell integrity and function.

Vitamin D functions to enhance protein synthesis and muscle cell growth, leading to hypertrophy and a greater number of type II muscle fibers [155]. These muscle fibers are particularly important, as they are the first to be recruited during falls [156]. Supporting these results, observational studies have reported that plasma vitamin D levels of 40–90 nmol/L are associated with enhanced musculoskeletal performance compared to those with serum levels below 40 nmol/L [157]. Evidence indicates that vitamin D deficiency is associated with impaired muscle function, which has been observed even in individuals without evident bone disease. [158]. These findings suggest that vitamin D status may be related to muscle strength through its roles in immune regulation, mitochondrial function, and muscle protein metabolism.

(3) Vitamin D-mediated regulation of mitochondrial activity and function in musculoskeletal tissues

In musculoskeletal tissues, vitamin D contributes to immune homeostasis and muscle function by regulating mitochondrial activity and oxidative stress. Vitamin D improves mitochondrial dynamics and reduces ROS generation, thereby promoting intracellular homeostasis and protecting against muscle damage [6]. Vitamin D deficiency has been reported to disrupt mitochondrial calcium uptake, leading to impaired metabolic homeostasis and muscle weakness, whereas 1,25(OH)_2_D enhances mitochondrial oxygen consumption and ATP production [159,160]. In addition, vitamin D supplementation attenuates oxidative damage in skeletal muscle by modulating antioxidant enzyme activity [161]. These effects are particularly important under inflammatory conditions, in which elevated pro-inflammatory cytokines and C-reactive protein impair mitochondrial function, and inflammation-induced inhibition of autophagy may further exacerbate mitochondrial dysfunction [162,163].

Mechanistically, vitamin D–VDR signaling regulates PGC-1α, a central mediator of mitochondrial biogenesis and redox homeostasis, while suppressing FOXO-dependent transcription of genes associated with muscle atrophy. Through these actions, vitamin D promotes a shift toward oxidative metabolism in skeletal muscle [7]. Vitamin D deficiency reduces expression of PGC-1α and insulin-like growth factor (IGF)-1 via VDR signaling, whereas vitamin D treatment upregulates VDR-mediated signaling and inhibits FOXO1 nuclear localization, transcriptional activity, and translocation, as demonstrated in the C2C12 cell line [164]. Moreover, in satellite cells and myocytes, the 1,25(OH)_2_D–VDR complex promotes differentiation and proliferation, while modulating the levels of mitofusin (MFN) 1/2, optic atrophy 1 (OPA1), and dynamin-related protein 1 (Drp1) proteins, thereby reducing oxidative stress and facilitating mitochondrial network remodeling [165].

(4) Regulation of bone–muscle interactions by vitamin D

Although definitive causal relationships remain incompletely understood, accumulating evidence supports the existence of molecular crosstalk and bidirectional communication between muscle and bone tissues [166]. This interplay is mediated by a complex network of signaling molecules, including myokines derived from muscle and osteokines produced by bone [167]. Such crosstalk is essential for maintaining musculoskeletal integrity and plays a key role in the pathogenesis of age-associated musculoskeletal disorders characterized by concurrent sarcopenia and osteoporosis [166].

Key mediators of the bone and muscle communication include muscle-derived signaling molecules including myostatin, irisin, IL-6, and IGFs, as well as bone-derived signaling molecules such as fibroblast growth factor (FGF)-23, Wnt3a, RANKL, and osteocalcin [166]. These molecules regulate anabolic and catabolic processes ensuring functional integration of muscle and bone within the musculoskeletal system [166].

Vitamin D is a central modulator within the signaling network between bone and muscle [168]. It facilitates bone–muscle communication primarily by attenuating chronic low-grade inflammation [169]. At the molecular level, vitamin D contributes to anti-inflammatory effects through suppression of TNF-α and IL-6, while enhancing anti-inflammatory signaling through the induction of Tregs and M2 macrophages [170]. These immunomodulatory effects promote muscle protein synthesis, inhibit bone resorption, and support overall musculoskeletal integration [170].

Given the complexity of bone–muscle interaction, elucidating the role of vitamin D within these interconnected pathways is critical for developing targeted approaches to prevent age-related musculoskeletal decline. Accordingly, maintaining adequate vitamin D status is essential for preserving bone and muscle health in aging populations.

### 4.2. Musculoskeletal Disorders: Biological Mechanisms and Clinical Evidence Related to Vitamin D

#### 4.2.1. Osteoporosis

Osteoporosis, according to the World Health Organization, is characterized by a BMD of the hip or lumbar vertebrae falls 2.5 SD or lower than the average BMD observed in a young adult reference population [171].

A major factor contributing to osteoporosis is chronic inflammation associated with aging [172]. As individuals age, they experience a sustained low-grade inflammatory response, referred to as inflammaging [173,174], which contributes to tissue dysfunction and the progression of age-related diseases [175]. In relation to bone health, inflammaging disrupts the normal bone remodeling cycle by enhancing bone resorption through increased osteoclast activity; while dysregulating osteoblast-mediated bone formation [176]. This imbalance ultimately results in decreased bone mass and a decline in bone quality, thereby increasing the risk of osteoporosis [176].

*(1) Mechanisms and* *in vitro and in vivo studies*

Vitamin D supports bone health through multiple mechanisms, including VDR-mediated transcriptional regulation and immunomodulatory effects through interactions with the immune system [177]. 1,25(OH)_2_D functions through interaction with VDR, which is found expressed in various cell types, including osteoclasts, osteoblasts, macrophages, and lymphocytes [178]. On a molecular level, vitamin D directly affects bone remodeling by modulating VDR-mediated gene expression in osteoclasts, thereby maintaining the homeostasis between bone resorption and synthesis [178]. Within bone tissue, vitamin D upregulates RANKL expression, which stimulates bone remodeling while simultaneously preventing osteoblast apoptosis [179]. Furthermore, vitamin D enhances osteoblast differentiation and function, promoting bone formation, while sustaining the equilibrium between osteoclasts and osteoblasts that is essential for skeletal integrity [180].

Vitamin D contributes to bone health by regulating inflammatory pathways. By activating VDR in immune cells and modulation of Tregs populations, vitamin D reduces chronic inflammation and protects against bone loss [178]. This immunoregulatory function is particularly significant given that inflammatory process plays a central role in the pathophysiology of osteoporosis [181]. Extensive research has examined the relationship between pro-inflammatory cytokines and bone metabolism [181]. In vitro studies have demonstrated that cytokines including IL-1, IL-6, and TNF-α regulate bone homeostasis [182]. Specifically, TNF-α and IL-1 act synergistically to promote osteoclast-mediated bone resorption in human through increased RANKL expression, where TNF-α requires IL-1 to achieve optimal osteoclast differentiation [183]. Animal model studies have indicated that these cytokines promote osteoporosis development by enhancing osteoclastogenesis and subsequent bone resorption [184]. Among pro-inflammatory markers, IL-6 has been extensively studied due to its induction by PTH [185]. However, in vitro studies have produced conflicting evidence regarding the impact of IL-6 on bone metabolism, indicating that it may promote both bone synthesis and resorption [186].

*(2) Human studies*

Numerous studies have explored the association between circulating vitamin D status and skeletal health in adults (Table 3). Higher levels of serum 25-hydroxyvitamin D have been linked to increased BMD, whereas lower vitamin D concentrations are consistently associated with reduced BMD as well as elevated PTH concentrations [187,188]. In these cross-sectional studies, associations were examined after adjustment for multiple potential confounding factors, including demographic characteristics, lifestyle-related variables, comorbidities, renal function, and total energy intake. In postmenopausal women, vitamin D insufficiency is associated with greater severity of skeletal disorders, including osteoarthritis-related symptoms and functional impairment [189]. Consistent with these results, findings from systematic reviews and meta-analyses demonstrated that higher serum 25-hydroxyvitamin D concentrations are associated with improved bone health in adult populations [190].

However, RCTs examining the impact of vitamin D supplementation on bone health have reported conflicting outcomes. Several RCTs have shown that vitamin D supplementation significantly increases BMD among postmenopausal women [191,192]. Consistently, a systematic review and meta-analysis demonstrated that combined treatment with an active vitamin D analogue and calcium was associated with improvements in myopathy associated with age-associated bone loss [155], indicating that vitamin D could exert beneficial effects on bone metabolism when administered alongside calcium. However, vitamin D supplementation in adults without deficiency has generally demonstrated no significant benefit for bone health [193], and vitamin D3 supplementation at elevated doses (4000 or 10,000 IU/day) has been linked to a decrease in BMD [193]. Additionally, a systematic review and meta-analysis encompassing 23 RCTs reported that supplementation with vitamin D alone did not result in a significant improvement in BMD in healthy adults or older individuals, suggesting that routine vitamin D supplementation in those without specific risk factors for vitamin D deficiency is not recommended for the prevention of osteoporosis [195].

#### 4.2.2. Sarcopenia

Sarcopenia is an age-associated, multifactorial condition characterized by the progressive loss of skeletal muscle mass and function. [196]. It has been consistently related to physical disability, reduced quality of life, and increased mortality [197]. Over time, diagnostic criteria have evolved to emphasize both muscle quantity and muscle strength and physical performance [198].

According to the 2019 consensus of the Asian Working Group for Sarcopenia (AWGS), sarcopenia is a progressive, age-related disorder of skeletal muscle, characterized by decreased muscle mass accompanied by either reduced muscle strength (handgrip strength < 28 kg in men and <18 kg in women) or impaired physical performance [198]. Muscle mass is evaluated using bioelectrical impedance analysis (BIA), with thresholds of appendicular skeletal muscle mass index set at <7.0 kg/m^2^ in men and <5.7 kg/m^2^ in women [198].

*(1) Mechanisms and in vitro and in vivo studies*

The relationship between vitamin D and muscle health involves multiple mechanistic pathways. Insufficient vitamin D levels lead to increased PTH secretion, which disrupts intracellular calcium homeostasis required for normal muscle contraction [199]. Furthermore, vitamin D modulates protein synthesis and muscle development through VDR activation in muscle tissue, thereby affecting gene expression patterns critical for myocyte proliferation and differentiation [200]. Previous evidence supports the significant role of VDR in muscle physiology has been well established through experiments demonstrating that VDR-deficient mice display reduced muscle fiber sizes, suggesting that the observed effects are facilitated through VDR signaling pathways [201].

Vitamin D plays a critical role in regulating immune and inflammatory responses, particularly in sarcopenia, a condition where chronic inflammation accelerates muscle deterioration and programmed cell death [202]. Through its interaction with VDR, vitamin D serves as an essential immunomodulatory factor influencing various immune cell populations [203]. Its anti-inflammatory effects are mediated through actions on macrophages and T lymphocytes, thereby mitigating muscle atrophy and functional deterioration [150]. Vitamin D enhances Tregs and anti-inflammatory macrophage populations, suppresses pro-inflammatory cells, including Th17 cells and cytotoxic T lymphocytes, thus preserving immune homeostasis [203]. Additionally, vitamin D reduces the synthesis of pro-inflammatory cytokines including IFN-γ, IL-1, IL-6, IL-12, IL-18, and TNF-α, which contribute to muscle protein breakdown and suppress anabolic processes [204].

In addition to its immunomodulatory role, vitamin D has been demonstrated to directly induce gene expression in hepatic cells, resulting in increased production of IGF-1, a hormone exhibiting anabolic and anti-inflammatory effects on muscle tissue [205]. Thus, vitamin D may alleviate sarcopenia by reducing inflammation and increasing IGF-1 production [206]. Given that apelin signaling is involved in numerous physiological functions, including suppression of inflammatory responses [207], this upregulation indicates potential therapeutic benefits of the supplementation with 1,25(OH)_2_D_3_ in mitigating age-associated muscle atrophy and cellular senescence [208].

Furthermore, vitamin D is involved in modulating muscle damage and facilitating regeneration processes [209]. Vitamin D deficiency induces mitochondrial impairment, lower ATP production, increased ROS generation, muscle atrophy, which may exacerbate adverse effects typically associated with muscle injury [209]. Skeletal muscle VDR expression correlates with serum 25(OH)D levels, whereas VDR and CYP27B1 exhibit minimal expression under homeostatic conditions [210]. Following muscle injury, 1,25(OH)_2_D significantly upregulates expression of VDR in satellite cells and central myonuclei, with localization to regenerating muscle fibers [211]. VDR is co-expressed with Pax7 in satellite cells [211], and both Pax7 and VDR levels increase rapidly following high-intensity, muscle-damaging exercise, indicating concurrent activation of myogenic repair mechanisms and vitamin D signaling pathways [212]. VDR signaling maintains satellite cell self-renewal capacity by inhibiting proliferation and promoting differentiation, while enhancing antioxidant capacity through increased mitochondrial biogenesis and reduced ROS production, thereby facilitating efficient muscle regeneration [209].

Furthermore, vitamin D treatment has been shown to suppress atrophy-associated proteins such as atrogin-1, and stimulate the expression of muscle-regulatory proteins, including MuRF1 and FOXO1 [213]. However, evidence investigating the effects of vitamin D supplementation on muscle performance has produced inconsistent findings. One study reported no alterations in type II muscle fiber proportions and no significant changes in muscle extension capacity or physical performance following vitamin D treatment [179].

*(2) Human studies*

Accumulating evidence suggests a relationship between vitamin D levels and muscle health (Table 4). In a prospective cohort study involving 1339 adults over three years, low serum 25(OH)D concentrations (<25 nmol/L) at baseline were associated with an increased risk of sarcopenia [208]. Additionally, other studies have reported that severe vitamin D deficiency is associated with higher fracture risk, reduced paraspinal muscle mass, and an increased prevalence of muscle-related disorders [208]. Consistent with these findings, a systematic review and meta-analysis of 30 RCTs involving 5615 adults reported that low vitamin D status was associated with impaired skeletal muscle outcomes and increased susceptibility to muscle-related disorders [214].

However, clinical trials examining the impacts of vitamin D supplementation on muscle health reported inconsistent results. In a randomized controlled trial involving 11 women with bone loss and muscle weakness, supplementation with 1–2 μg of 1α-hydroxycholecalciferol combined with 1 g of calcium daily for a period of 3–6 months improved myopathy associated with age-related bone loss [155]. Similarly, an RCT conducted in 38 adults reported that daily supplementation with 3200 IU per day of vitamin D_3_ over 5 weeks enhanced muscle protein synthesis signaling, suppressed muscle atrophy pathways, and improved mitochondrial oxidative capacity compared with placebo [207]. In contrast, other RCTs have reported no significant effects of vitamin D supplementation on muscle function or strength [215,216]. Moreover, a systematic review and meta-analysis including 10 clinical trials reported that vitamin D supplementation was not associated with significant improvements in muscle mass, muscle strength, or overall physical performance [217]. These inconsistent results may result from heterogeneity in study design, including variations in participant age, vitamin D levels at baseline, supplementation dose, and intervention duration.

**Table 4:** Vitamin D and muscle diseases.

Vitamin D status and muscle diseases			
Study design	Status of participants	Outcome	Reference
Prospective cohort study	1339 adults from the Longitudinal Aging Study Amsterdam, 55–85y, follow-up 3 y	Low baseline 25(OH)D (<25 nmol/L) was associated with an increased risk of sarcopenia compared to individuals with high levels (>50 nmol/L), with a 2.57-fold increase based on grip strength and a 2.14-fold increase based on muscle mass	[208]
Prospective cohort study	1604 women from the OPRA study, 75 y, mean follow-up 3 y	Severe deficiency in vitamin D (<20 ng/mL) was associated with higher fracture risk, possibly due to reduced physical activity and impaired postural stability	[218]
Cross-sectional study	976 adults from the InCHIANTI study, mean age of 75 y	Vitamin D levels was negatively correlated with poor physical performance	[219]
Cross-sectional study	91 women, age 60–69 y	Vitamin D deficiency was linked to decreased paraspinal muscle mass and greater fatty infiltration	[220]
Systematic review and meta-analysis	30 clinical trials involving 5615 adults, mean age 61 y	Low vitamin D status was related with reduced muscle function and elevated risk for muscle-related disorders	[214]
Effect of vitamin D supplementation on muscle diseases			
Study design	Status of participants	Outcome	Reference
RCT	11 women with bone loss and muscle weakness, 65–81 y Received 1–2 μg 1α-hydroxycholecalciferol + 1 g of calcium, 3–6 mo	Combined administration of the active form of vitamin D analogue (1α-hydroxycholecalciferol) and calcium can improve myopathy associated with age-related bone loss	[155]
RCT	38 adults with chronic low back pain (19 men and 19 women), 45–52 y Received 3200 IU/day vitamin D3 or placebo, 5 wk	Vitamin D supplementation enhanced muscle protein synthesis signaling, suppressed muscle atrophy pathways, and improved mitochondrial oxidative capacity	[207]
RCT	89 adults, age over 65 y Received 0.25 μg of vitamin D3 twice daily or placebo, 6 mon	Oral administration of vitamin D did not improve muscle strength in older people	[215]
RCT	243 adults, age 78–80 y Four groups: Exercise + Vitamin D3 Exercise + Placebo, No Exercise + Vitamin D3 No Exercise + Placebo. Oral Vitamin D3 supplementation (300,000 IU/day), 10 wk	Vitamin D supplementation, whether alone or combined with exercise, showed minimal or no effect on muscle strength, function, or fall prevention	[216]
Systematic review and meta-analysis	10 clinical trials involving 1862 adults, age 58–80 y	Vitamin D supplementation showed no benefit on muscle mass, muscle strength, or overall physical performance	[217]

25(OH)D, 25-hydroxyvitamin D; OPRA, Osteoporosis Prospective Risk Assessment study; InCHIANT, Invecchiare in Chianti study; Randomized controlled trial, RCT.

## 5. Conclusions

**Figure 1:**
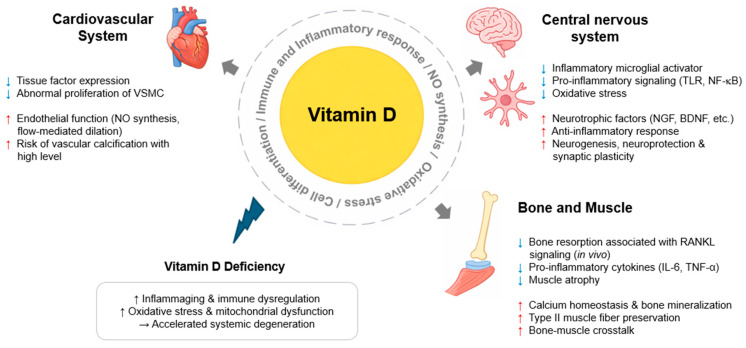
Vitamin D-mediated regulation in cardiovascular, central nervous, and musculoskeletal systems. Blue arrows indicate a decrease, and red/black arrows indicate an increase. Abbreviations: BDNF, brain-derived neurotrophic factor; NF-κB, nuclear factor-κB; NGF, nerve growth factor; NO, nitric oxide; TLR, toll-like receptor; VSMC, vascular smooth muscle cell.

Vitamin D deficiency has been consistently associated with increased risks of cardiovascular, neurodegenerative, bone, and muscle diseases through shared mechanisms involving cellular and mitochondrial dysfunction, immune dysregulation, and increased oxidative stress. In addition, vitamin D exerts disease-specific effects across different tissues. In cardiovascular tissues, vitamin D has been shown to regulate endothelial function and VSMC foam cell formation. In the CNS, vitamin D influences neuroinflammatory and oxidative stress responses in neurons and glial cells, potentially contributing to pathological processes in AD and PD. In bone and muscle tissues, vitamin D supports immune homeostasis, mitochondrial function, and bone–muscle crosstalk through myokine-osteokine signaling (Figure 1).

However, despite substantial mechanistic evidence, clinical trials of vitamin D supplementation in cardiovascular, neurodegenerative, and musculoskeletal diseases have reported heterogeneous and sometimes null or adverse outcomes. These inconsistencies suggest that the biological effects of vitamin D are highly context- dependent and do not uniformly translate into clinical benefit across heterogeneous human populations. This variability may reflect differences in serum 25(OH)D thresholds used to define deficiency, as well as individual factors such as baseline vitamin D status, frailty, physical activity, nutritional status, comorbidities, and disease stage. Accordingly, clinical outcomes should be interpreted with careful consideration of the interacting health-related factors that may influence observed associations, rather than being viewed as evidence of uniform or context-independent effects of vitamin D supplementation.

Furthermore, current evidence indicates that vitamin D supplementation may be beneficial for individuals with deficiency or those at high risk; however, these findings cannot be directly applied to routine supplementation for degenerative diseases. Therefore, further well-designed longitudinal and interventional studies using targeted approaches, such as biomarker-guided supplementation and disease-stage-specific interventions, are required to delineate the conditions under which vitamin D may serve as an effective preventive or adjunctive strategy for degenerative disorders.

## Figures and Tables

**Table 1:** Vitamin D and Dementia including Alzheimer’s disease (AD).

Vitamin D status and dementia			
Study design	Status of participants	Outcome	Reference
Meta-analysis of dose–response	Seven prospective cohort studies and one retrospective cohort study (*n* = 28,354; dementia cases = 1953; AD cases = 1607)	Significantly higher pooled HR of dementia and AD for vitamin D deficiency (<10 ng/mL) but not in insufficiency (10–20 ng/mL).	[93]
Meta-analysis of prospective studies	Nine studies for risk of dementia and four studies for risk of AD	Significantly 1.42 times higher risk for dementia and 1.57 higher risk for AD.	[90]
Meta-analysis	Twelve prospective cohort studies and four cross-sectional studies	Significantly higher pooled HR (1.32 and 1.48) of dementia and AD for vitamin D deficiency (<20 ng/mL)	[94]
Meta-analysis of dose–response	Ten cohort studies, with 28,640 participants	Significant inverse association between 25(OH)D levels and the risk of dementia and AD. Linear dose–response relationship between 25(OH)D levels and reduced risk of dementia or AD.	[95]
Effect of vitamin D supplementation on dementia			
Study design	Status of participants	Outcome	Reference
RCT	AD patients (*n* = 210); vitamin D 800 IU/day vs. placebo (starch), 12 months	Daily oral vitamin D (800 IU/day for 12 months: improved cognitive function and reduced Aβ-related biomarkers.	[96]
RCT	Community-dwelling older adults (*n* = 63; age > 60 years; 32 with mild–moderate disease); high- vs. low-dose vitamin D (1000 IU/day) for 8 weeks, followed by intranasal insulin.	High-dose vitamin D showed no additional benefit over low-dose vitamin D for cognition or disability in patients with mild-moderate AD	[97]
RCT	Finnish Vitamin D Trial participants (*n* = 2492; dementia-free at baseline); vitamin D_3_ 1600 or 3200 IU/day for up to 5 years	Five-year supplementation with medium- or high-dose vitamin D did not reduce the risk of dementia.	[98]
Meta-Analysis	Nine RCTs with 2345 participants	No significant preventive effect of vitamin D supplementation on AD, with no improvements in verbal fluency, verbal memory, visual ability, or attention scores.	[99]

Alzheimer’s disease, AD; Hazard ration, HR; Randomized controlled trial, RCT.

**Table 2:** Vitamin D and Parkinson’s disease (PD).

Vitamin D status and PD			
Study design	Status of participants	Outcome	Reference
Meta-analysis	Seven observational studies (1008 patients and 4536 controls)	Patients with vitamin D deficiency or insufficiency: significantly increased PD risk (OR: 1.5, 95% CI 1.1–2.0; OR: 2.2, 95% CI 1.5–3.4)	[120]
Meta-analysis	Eight studies (five case–control, one cohort, and two RCTs)	Vitamin D deficiency or insufficiency: significantly increased PD risk (OR: 2.55, 95% CI 1.98–3.27; OR: 1.77, 95% CI 1.29–2.43) Sunlight exposure (15 min/week): significantly reduced PD risk (OR: 0.02; 95% CI 0.00–0.10).	[117]
Meta-analysis	Twenty studies (2866 patients with PD and 2734 controls)	Lower serum vitamin D levels in PD patients compared with controls Serum vitamin D levels were inversely correlated with PD severity (r = −0.55, 95%CI −0.73, −0.29) and UPDRS III scores (r = −0.36, 95%CI −0.53, −0.16), but showed no association with disease duration or patient age	[121]
Meta-analysis	Seven studies (5690 patients with PD and 21,251 matched controls).	Vitamin D deficiency and insufficiency were associated with higher PD risk compared with matched-controls (OR: 2.08, 95% CI: 1.63 to 2.65, and 1.29, 95% CI: 1.10 to 1.51). Outdoor work was associated with a reduced risk of PD (OR: 0.72, 95% CI: 0.63 to 0.81)	[122]
Umbrella review	14 meta-analyses were included in the meta-review	Lower serum vitamin D and B12 levels in patients with PD.	[123]
Effect of vitamin D supplementation on PD			
Study design	Status of participants	Outcome	Reference
Meta-analysis	Eight studies (five case–control studies, one cohort study, and two RCTs)	Vitamin D supplementation showed no significant effect on motor function in patients with PD.	[117]
Meta-analysis	Seven studies (5690 PD patients and 21,251 matched controls).	Vitamin D supplementation was associated with reduced risk of PD (OR: 0.62, 95% CI: 0.35 to 0.90).	[122]
RCT	30 PD patients with vitamin D deficiency were received vitamin D3 (*n* = 15) or placebo (*n* = 15) for three months.	Vitamin D3 supplementation significantly increased 25(OH)D3 levels, reduced Th17 cells, and elevated Tregs. Vitamin D supplementation improved motor function.	[124]
RCT	Patients with PD (*n* = 114) were randomly assigned to vitamin D3 supplementation (*n* = 56; 1200 IU/d) or a placebo (*n* = 58) for 12 months	Vitamin D supplementation was associated with reduced deterioration of the HY stage, particularly in patients with *FokI* TT or CT genotypes.	[125]
RCT	Patients with PD were randomly assigned to receive vitamin D3 supplementation (*n* = 13) or placebo (*n* = 16)	Vitamin D supplementation significantly improved Up and Go and the 6 MWT, with no effect on serum hs-CRP levels.	[126]

Hoehn and Yahr, HY; min walk test, MWT; Parkinson’s disease, PD; Randomized controlled trial, RCT; regulatory T cells, Tregs; Unified Parkinson’s Disease Rating Scale III, UPDRS III.

**Table 3:** Vitamin D and bone diseases.

Vitamin D status and bone diseases			
Study design	Status of participants	Outcome	Reference
Cross-sectional study	1319 adults (643 men and 676 women), 65–88 y	Higher serum 25(OH)D concentrations above 50–60 nmol/L were linked to high BMD at hip, trochanter, and total body BMD; low 25(OH)D was correlated with decreased BMD and elevated PTH	[187]
Cross-sectional study	203 women, 18–35 years	Serum 25(OH)D levels were negatively associated with PTH and osteocalcin	[188]
Cross-sectional study	54 postmenopausal women, age over 45 y	Vitamin D deficiency is significantly associated with increased severity, flares, and functional disability linked to knee osteoarthritis among postmenopausal women	[189]
A systematic review and meta-analysis	35 studies, mean age of 20–59 y	Serum 25(OH)D levels were positively associated with bone health in adult individuals	[190]
Effect of vitamin D supplementation on bone diseases			
Study design	Status of participants	Outcome	Reference
RCT	70 postmenopausal women with osteoporosis, 51–69 y Received either 0.5 μg/day vitamin D3 combined with 1000 mg/day of calcium or placebo, 6 mo	Combination therapy with calcitriol and calcium significantly higher BMD and lower IL-1 and TNF-α levels in postmenopausal women with osteoporosis compared to those in the placebo group	[191]
RCT	305 postmenopausal women, 60–70 y Received 400 or 1000 IU/day Vitamin D_3_ vs placebo, 1 y	Vitamin D supplementation significantly increased bone density primarily in adults with vitamin D deficiency (25(OH)D ≤ 30 nmol/L)	[192]
RCT	452 community-resident adults, 50–84 y Received 100,000 IU/month of Vitamin D3, 2 y	No significant change in BMD was observed following vitamin D supplementation, although an increase in BMD was found in those with baseline 25(OH)D ≤ 30 nmol/L	[193]
RCT	311 healthy adults, mean age 62 y Received 4000 IU/ day or 10,000 IU/day compared to 400 IU/day, 3 y	Vitamin D supplementation at doses of 4000 IU/day or 10,000 IU/day resulted in significantly lower radial BMD	[194]
A systematic review and meta-analysis	23 clinical trials involving 4082 adults, mean age of 59 y	Combined treatment with the active vitamin D analogue (1α-hydroxycholecalciferol) and calcium can improve myopathy associated with age-related bone loss	[155]
A systematic review and meta-analysis	23 studies involving 4082 adults, mean age of 59 y	Vitamin D supplementation did not show a significant change in bone mineral density in healthy adults and older individuals	[195]

25(OH)D, 25-hydroxyvitamin D; Bone mineral density, BMD; Parathyroid hormone, PTH; Randomized controlled trial, RCT.

## Data Availability

No new data were created or analyzed in this study. Data sharing is not applicable to this article.
